# A Selfish Genetic Element Influencing Longevity Correlates with Reactive Behavioural Traits in Female House Mice (*Mus domesticus*)

**DOI:** 10.1371/journal.pone.0067130

**Published:** 2013-06-24

**Authors:** Yannick Auclair, Barbara König, Anna K. Lindholm

**Affiliations:** Institute of Evolutionary Biology and Environmental Studies - Animal Behaviour, University of Zurich, Zurich, Switzerland; VIB & Katholieke Universiteit Leuven, Belgium

## Abstract

According to theory in life-history and animal personality, individuals with high fitness expectations should be risk-averse, while individuals with low fitness expectations should be more bold. In female house mice, a selfish genetic element, the *t* haplotype, is associated with increased longevity under natural conditions, representing an appropriate case study to investigate this recent theory empirically. Following theory, females heterozygous for the *t* haplotype (+/*t*) are hypothesised to express more reactive personality traits and be more shy, less explorative and less active compared to the shorter-lived homozygous wildtype females (+/+). As males of different haplotype do not differ in survival, no similar pattern is expected. We tested these predictions by quantifying boldness, exploration, activity, and energetic intake in both +/*t* and +/+ mice. +/*t* females, unlike +/+ ones, expressed some reactive-like personality traits: +/*t* females were less active, less prone to form an exploratory routine and tended to ingest less food. Taken together these results suggest that differences in animal personality may contribute to the survival advantage observed in +/*t* females but fail to provide full empirical support for recent theory.

## Introduction

In a wide range of taxa, it has been shown that individuals from the same population differ consistently in their behaviour. The concept of animal personality applies to behavioural differences that are consistent through time and situations [1], [2], [3]. Often, these behavioural traits are correlated within or across contexts and are referred to as behavioural syndromes [4], [5], [6]. For instance, “proactive” individuals, in contrast to “reactive” individuals, have higher activity levels and a higher metabolic rate, are more exploratory and risk-prone (or bold), and faster to establish routines [7], [8], [9], [10], [11]. How animal personalities evolved within populations still remains unclear, especially because behavioural plasticity could be seen as an optimal way to cope with fluctuating environments [12].

Life-history theory provides a framework for investigating the evolution of animal personalities [13], [14], [15]. Animal personality can have a profound influence on life-history traits like growth, fecundity and survival [15], [16], [17]. Using evolutionary models, Wolf and co-workers [14] demonstrated that life-history tradeoffs promote the evolution of animal personalities. Individuals varying in exploration behaviour inhabited a low-quality resource habitat for a year at the end of which they could stay for a second year or move to a high-quality resource habitat. Superficial explorers, that evolved high levels of boldness in risky games ( = proactive), invested more in current reproduction. Conversely, those that invested more in future reproduction were careful explorers, that evolved low levels of boldness in the same risky games ( = reactive). These models therefore predict that individuals with different fitness expectations express different personality traits, here exploratory behaviour. The authors concluded that individuals with high expectations of future fitness, who have much to lose and for whom life is valuable, should be more cautious than individuals with low expectations.

Concurring with model predictions, recent evidence shows that individuals expressing reactive personality traits have a lower basal metabolic rate and therefore lower energetic needs [10], [11]. Metabolism of reactive individuals could allow them to survive longer by saving more energy than proactive individuals, especially when foraging involves risk-taking. For instance, a personality implying less risk-taking behaviour and conserving energy would favour survival [16], [18]. Thus, long-lived individuals should express a reactive-like personality whereas individuals characterized by a low life expectancy should express a proactive-like personality [14].

The *t* haplotype, also called the “*t* complex”, a naturally occurring genetic variant in the house mouse (*Mus domesticus*), provides an appropriate case study to investigate this hypothesis and hence fill the gap of empirical data. The *t* haplotype is a selfish genetic element, consisting of many linked genes, showing drive [19]. Its main known fitness effect is a reduction in litter size in matings between heterozygotes due to a recessive lethal allele [20]. Recently, *t* related effects on life-history have been documented. In a free-living population of house mice, female heterozygotes (+/*t*) live longer than homozygous wildtype females (+/+), with a 30% viability advantage [21]. No difference in survival was found between +/+ and +/*t* males. Although no information is yet available on whether life expectancy positively correlates with fitness in wild house mice, mean life expectancy has been reported to be 100–150 days [22], [23] whereas generation time is about 270 days [24]. This indicates that many mice die before they successfully reproduce, thus suggesting that a higher life expectancy could improve the chance to reproduce.

Following theory on the evolution of life-history and personality [13], [14], we hypothesize that reactive personality traits co-evolved with the *t* haplotype. We therefore assessed personality traits in mice of both sexes and genetic backgrounds. We predicted that +/*t* females, characterized by a high survival rate, should express “reactive-like” personality traits and therefore be more shy, less active and less explorative compared to +/+ females, characterized by a lower survival rate. Moreover, we compared the propensity of +/+ and +/*t* to form routine as it has been shown to reflect individuals’ ability to use information on their environment and then adapt to its potential changes [7], [9], [25]. House mice travel their territory daily, covering and marking the same routes repeatedly. Through these routines, mice acquire highly habitual responses, which they can perform rapidly and with minimal sensory input [26]. As proactive individuals form routines faster than reactive individuals, we expect +/+ females to form such routines faster than +/*t* females. Finally, as an index of energy intake we monitored food consumption, expecting that reactive individuals, here +/*t* females, ingest less food compared with proactive individuals, here +/+ females [10], [11]. No differences were expected between males of different haplotypes as they have a similar survival rate.

## Methods

### Ethics Statement

Animal use and experimental design were approved by the Veterinary Office Zürich, Switzerland (Kantonales Veterinäramt Zürich, no. 97/2009).

### Study Subjects

We used 82 sexually mature but non-breeding house mice (more than six weeks old; mean age ± SE = 184±10 days) which were laboratory born F2 and F3 descendants of wild-caught individuals from the same population in the vicinity of Zürich as the one in which longevity differences were reported [21]. We tested a total of 41 females (20 were +/+ and 21 were +/*t*) and 41 males (20 were +/+ and 21 were +/*t*) randomly selected from offspring of our breeding stock. No significant difference in age was observed between +/+ and +/*t* mice of the same sex (females: *t_39_* = 0.03, *p* = 0.973; males: *t_39_* = 0.84, *p = *0.408). Males were younger than females (*t_80_* = 4.02, *p*<0.001), because high aggression among males meant that they could not long be housed in groups. All individuals were in good condition for the entire duration of the study.

### Housing

All mice were singly housed in Macrolon Type II cages (267×207×140 mm), beginning 5 days before the first behavioural test. Each cage contained standard animal bedding (Lignocel Hygienic Animal Bedding, JRS), an empty toilet paper roll and some paper towel as hides and nest building material. Food (laboratory animal diet for mice, Provimi Kliba SA, Kaiseraugst, Switzerland) and water were provided *ad libitum*. Animals were kept under standardized laboratory conditions at a temperature of 22°C ±3°C with a relative humidity of 50–60% and on a 14∶10 light:dark cycle with a 1 h sunrise and dusk phase at the beginning and end of the light phase.

### Body Weight

Mice were weighed twice at a 7-day interval with the first measurement the day before the first behavioural test and the second on the day following the end of the first series of behavioural tests. We did not observe significant changes in body weight (*t_81_* = 1.69, *p* = 0.095). As the two measurements were highly repeatable (*R = *0.95, *F_81,82_* = 40.52, *p*<0.001), we used the mean.

### Genotype Determination

An individual ear tissue sample was collected from all males and females at least one week before testing. DNA was isolated and amplified at the *Hba-ps4* locus, a marker containing a 16-bp *t* haplotype specific insertion [27]. PCR products were electrophoresed using an ABI 3730x1 and visualized using Genemapper 4.0 software (Applied Biosystems) to determine genotype at the *t* locus.

### Schedule for the Assessment of Personality Traits

For breeding convenience this study was realized in two sessions. The first session took place in February – March whereas the second session took place in July – August. Each behavioural test was performed twice with a seven day interval to check for individual consistency through time [28], [29], [30]. Exploration tests were however replicated after nine days because of a time constraint. Activity and boldness tests were performed in the morning (from 8∶00 to 11∶00), whereas the first assessment of exploratory behaviour was performed in the afternoon (15∶00 to 18∶00) and the replicate in the morning. All behaviour tests lasted ten minutes, with the observer standing immobile at a one meter distance. As the activity and boldness tests were performed using the home cage of the mice, the stress induced by the procedures was very limited. Within a three minute acclimation period the mice were very calm and were observed grooming themselves. A single mouse was involved in only one experiment per day and had one day free after each behavioural test. The behavioural tests were run blindly with regard to the genotype of the mice.

### Activity

To measure individual activity, we removed nest material and the paper roll from the home cage to facilitate observations. We replaced the cage lid by a Plexiglas lid with a grid drawn on it to uniformly split the cage widthwise into three equal parts. After a three minute acclimation period, the observer recorded the number of times a mouse crossed the lines with all four paws for ten minutes. We then calculated an activity score following previous common procedures [31], [32].

### Exploration

**Figure 1:**
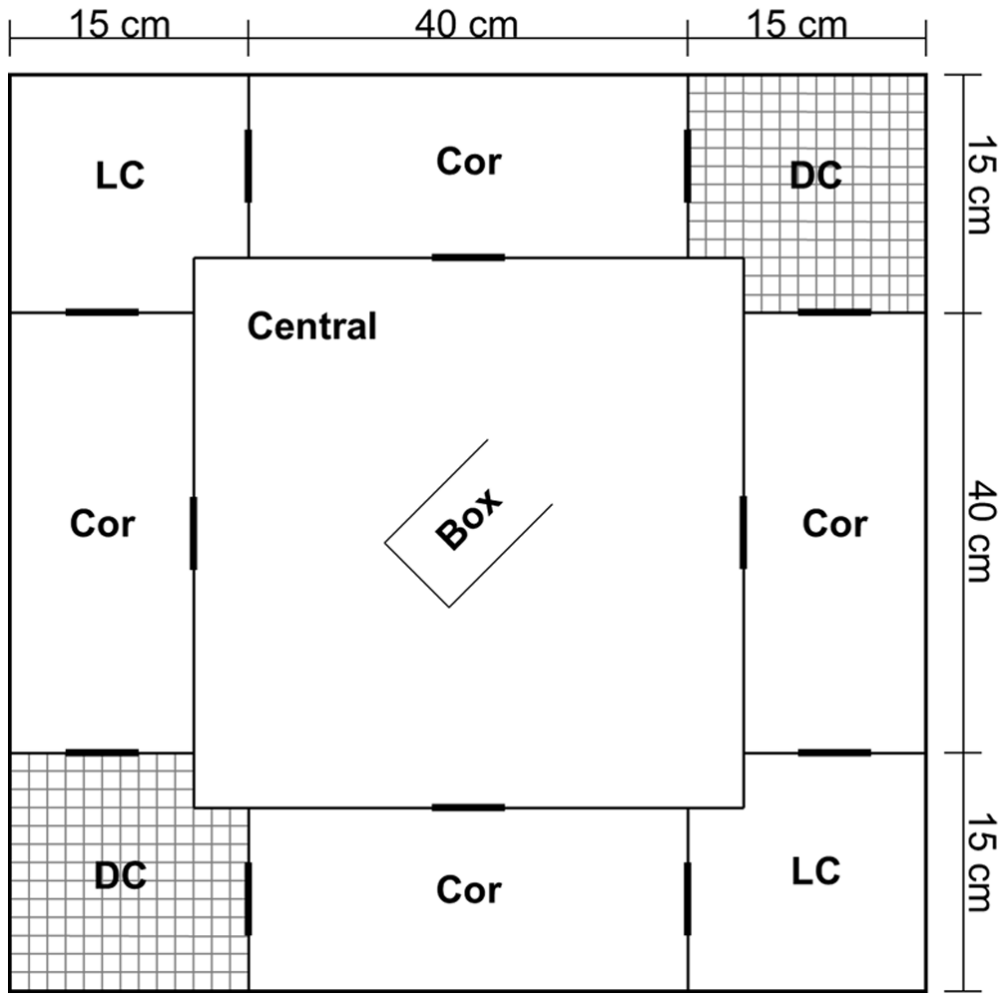
Concentric square field cage to test exploratory behaviour. CC = Covered Corners; Cor = Corridors; UC = Uncovered Corners; Central = Central Compartment; Box = dark box in which mice were transferred to the experimental cage. The holes, drawn in bold in the figure, that connect corridors with corners are 10 cm above the ground whereas the holes that connect the central area with the corridors are 1.5 cm above the ground.

Exploratory behaviour was assessed in a concentric square field cage representing an arena composed of nine compartments, a central part surrounded by four corridors joined alternatively by covered and uncovered corners [33], [34] (Figure 1). After each trial, the apparatus was cleaned with acetone to remove scent marks [35]. A focal mouse was transferred in a small dark box from its home cage to the apparatus to reduce stress before the beginning of the test. The door of the box was aimed at the direction of a covered corner in the first trial and at the direction of an uncovered corner in the replicate. The sliding door of the box was opened by remote control (using a string), and latency time to leave the box, time needed to enter each compartment, and total number of visits to compartments were recorded. For convenience latencies were subtracted from the total duration of the test (600 seconds) such that highly explorative individuals, characterized by short latencies, received a high value.

### Boldness

Boldness was assessed in a classical olfactory test realized with three Macrolon type II cages connected by tubes [36], [37]. We connected a central cage to two cages, one at each side. The central cage was filled with bedding from the home cage of the individual tested. The two other cages were filled with either unused cat bedding for one or with soiled cat bedding for the other (Cat’s Best Öko Plus, Qualipet). The soiled cat bedding had been used by a domestic cat during one week before the experiment. Cats represent a natural predator against which mice should have evolved avoidance mechanisms [38], [39]. Following Dickman & Doncaster [40], mice should be able to assess the presence of predators indirectly through olfactory cues and avoid areas with predator’s faeces or urine. Our setting thus represents two identical areas, one of which has apparently been visited by a natural predator, allowing a test of boldness in the face of predator cues [41], [42], [43]. This procedure avoids a repeated exposure to a real predator, known to be highly stressful for mice [34].

Focal individuals were released in the central cage and kept there for a three minute acclimation period. Removable wire mesh partitions closed the tubes, allowing odour identification of the neighbour cages. At the start of the trial, partitions were removed and the time spent and the number of visits to each cage containing each type of cat bedding were recorded for ten minutes. The mice gave significantly more visits to (*t_81_* = −3.25, *p* = 0.002) and spent significantly more time (*t_81_* = −2.88, *p* = 0.005) in the cage filled with unused cat bedding than in the cage filled with soiled cat bedding.

### Propensity to Form Routine

Routine formation is usually measured by changing a familiar environment that has been experienced repeatedly and subsequently testing how quickly individuals react to this environmental change [7], [9], [44]. The propensity to form routine can be indirectly measured by the magnitude of the increase in the performance of a given behaviour between the replicated trials of the same test. Following this idea, we quantified the propensity to form routine as the difference between the performance measured at the second trial and the performance measured at the first trial.

### Food Consumption

Food consumption was only recorded for the 48 mice taking part in the second session because of a time constraint at the end of the first session. This sub-sample was composed of 23 females (12+/*t* and 11+/+) and 25 males (9+/*t* and 16+/+). During two consecutive weeks, one month after all behavioural experiments were carried out, the quantity of pellets eaten by the mice was recorded at the same time of day. On day 1 the food holder was cleaned and filled with new pellets of known quantity (weighed on an electronic balance, Sartorius BL 1500 S, with 0.01 g. precision). At day 7 and 14, uneaten pellets were removed for weighing, and at day 7 replaced with new pellets. We checked daily if pieces of pellets had fallen through the feeder grid into the bedding. When found, they were removed and weighed. Food consumption was repeatable between the two weeks (intra-class correlation coefficient: *R* = 0.44, *F_47,48_* = 2.55, *p*<0.001).

### Statistical Analyses

Statistical tests were carried out using R version 2.13.1 (R development core team 2011). Numbers of visits in the cage containing soiled cat bedding, number of visits in the cage containing clean cat bedding, and total number of visits in all compartments of the exploration apparatus test were square-root transformed, while activity scores, the time needed to explore all the compartments in the exploration test, and quantity of food eaten were log-transformed to satisfy normality.

We tested the influence of individual identity, the genetic background, sex, body weight, session and trial on the variables measured using linear mixed effect models. Interactions between genetic background and sex, trial and sex, trial and genetic background, and between trial, genetic background and sex were also included.

Individual identity was defined as a random effect to assess individual consistency (repeatability) while all other variables were defined as fixed effects. Significance of the random effect was determined by likelihood ratio tests while fixed effects were tested using F tests [45]. We also used ANOVA-based intra-class correlation coefficients (*R*) to quantify individual consistency between the two trials of each behavioural test [46], [47]. A significant effect of trial in the mixed effect models described above revealed a propensity to form routine. Potential effects of genetic background, sex or their interaction on routine formation were therefore assessed by the effect of the interactions involving trial in the same mixed effect models.

Multiple correlations between the personality traits showing individual consistency enabled us to check for correlations between personality traits. To avoid type I errors, we followed the Benjamini & Hochberg procedure that also reduced type II errors by controlling false discovery rate [48], [49]. Beforehand, the number of movements in the activity test, total number of visits to compartments in the exploration test, and the number of visits to cages containing clean and soiled cat bedding were averaged and then standardized (for each session separately) to control for the “session” effect found in the mixed effect models. For each trial the standardized variables are thus defined by an identical mean (equal to 0) and standard deviation (equal to 1).

Food consumption (total food consumed over two weeks) was normally distributed and was analysed using a general linear model to determine the influence of the genetic background, sex, body weight and their interactions. Non significant interactions (*p*<0.05) were dropped from the full model by a backwards stepwise procedure, following Crawley [45].

## Results

### Individual Consistency

The number of movements during the activity test, the total number of visits to and the time needed to explore all the compartments in the exploration test, and the numbers of visits to the cage containing soiled cat bedding during boldness tests were consistent within an individual through time (Table 1). These variables were therefore used to test for behavioural syndromes.

**Table 1:** Individual consistency of the behavioural variables assessed twice at a one-week interval, estimated firstly from mixed model analysis accounting for genetic background, body weight, sex, session, trial and interactions, and secondly from ANOVA-based intra-class correlation coefficients.

Personality traits	Parameters	ID as a random effect		Intra-class correlation coefficients		
		*Likelihood ratio*	*p*	*R*	*F_81,82_*	*p*
**Activity**	number of movements	36.05	<0.0001	0.73	6.49	<0.001
**Boldness**	number of visits to soiled cat bedding	7.36	0.007	0.31	1.91	0.002
	time spent in soiled cat bedding	0.01	0.999	−0.08	0.85	0.759
**Exploration**	total number of visits in compartments	23.49	<0.0001	0.43	2.53	<0.001
	time needed to visit all compartments	23.94	<0.0001	0.48	2.82	<0.001

### Personality Traits

**Figure 2:**
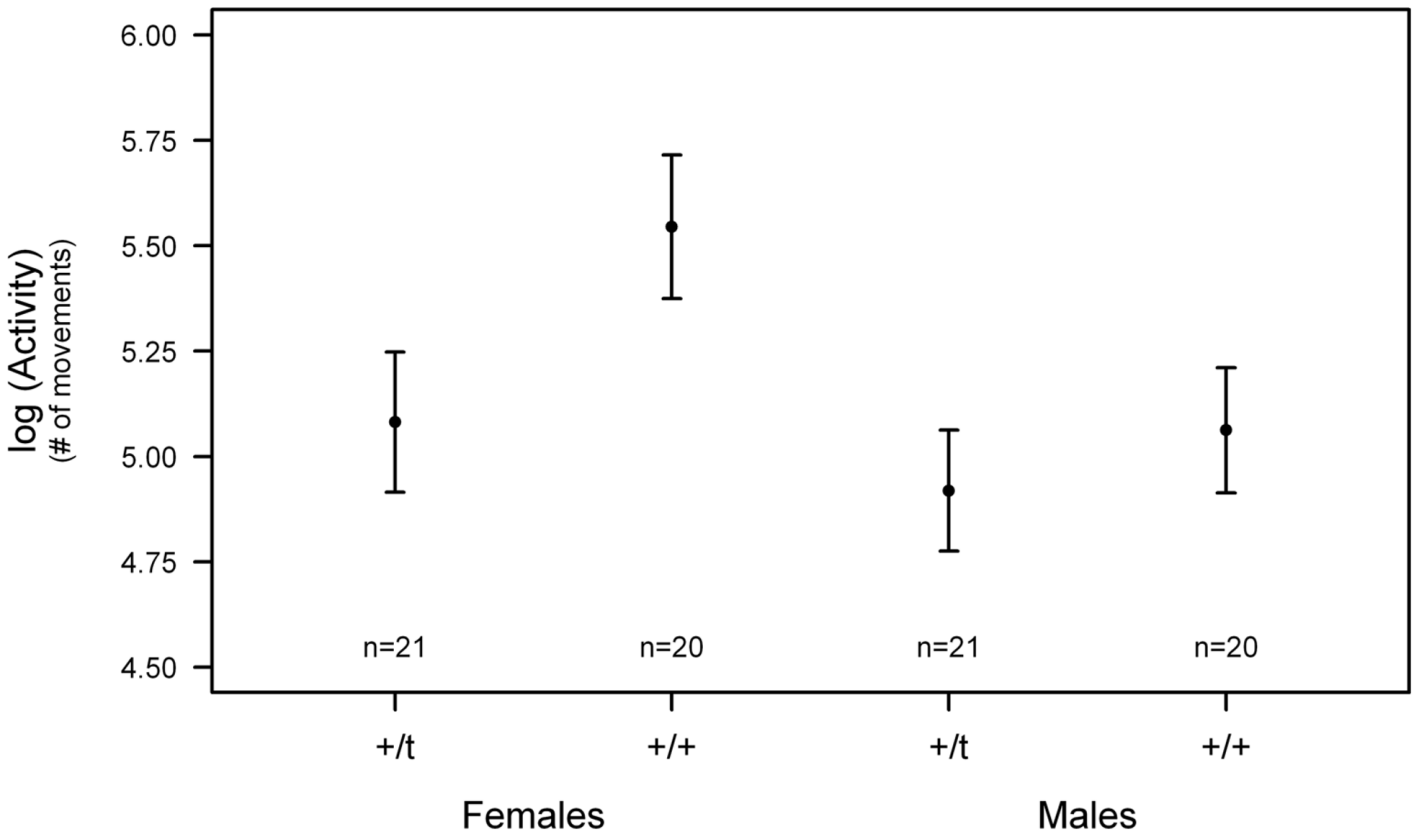
Effect of the genetic background and sex on activity score (mean ± standard errors predicted by mixed effect model).

The analyses of the influence of the genetic background, sex, and body weight on the personality traits showed that both the *t* haplotype, sex and their interaction had a significant effect on basic activity (Table 2). +/*t* females were less active than +/+ females, and females were in general more active than males (Figure 2). None of the personality traits measured in the boldness and exploration tests were influenced by the genetic background, sex or their interaction (Table 2). Body weight did not have any significant effect in any of the personality traits except for the total number of visits in the exploration test (Table 2).

**Table 2:** Mixed model analysis of the personality traits showing individual consistency.

	Personality traits							
	Activity		Boldness		Exploration			
	# movements		# visits to soiled bedding		# visits in compartments		time needed to visit compartments	
	*F*	*p*	*F*	*p*	*F*	*p*	*F*	*p*
Genetic background	5.37	0.023	0.03	0.853	1.76	0.188	0.21	0.650
Sex	5.88	0.018	1.91	0.171	1.14	0.289	0.42	0.517
Body weight	2.69	0.105	0.94	0.337	5.58	0.021	0.04	0.847
Session	30.21	<0.001	27.40	<0.001	78.23	<0.001	0.11	0.739
Trial	1.10	0.298	25.31	<0.001	75.77	<0.001	29.68	<0.001
Genetic background : Sex	5.20	0.025	0.09	0.770	1.04	0.312	2.51	0.118
Sex : Trial	0.07	0.787	0.10	0.754	0.02	0.876	0.78	0.380
Genetic background : Trial	0.02	0.877	1.40	0.241	0.66	0.418	0.02	0.881
Genetic background : Sex : Trial	0.18	0.671	1.12	0.293	6.52	0.013	0.29	0.592

### Propensity to Form Routine

No propensity to form routine was observed in the activity test as mice showed similar activity scores between the first and the second trial (Table 2). However, during the boldness test the number of visits increased during the second trial to both the cage with soiled cat bedding (1^st^ trial (mean ± SE): 5.5±0.6, 2^nd^ trial: 8.4±0.6) and the cage with clean cat bedding (1^st^ trial (mean ± SE): 6.6±0.6, 2^nd^ trial: 9.0±0.6) (Table 2). During the exploration test, the total number of visits to the compartments increased between the first and the second trial (1^st^ trial: 23.1±2.7, 2^nd^ trial: 45.9±4.8) whereas the time needed to explore all the compartments decreased (1^st^ trial: 560±12 sec., 2^nd^ trial: 468±22 sec.), both suggesting a propensity to form an exploratory routine (Table 2).

**Figure 3:**
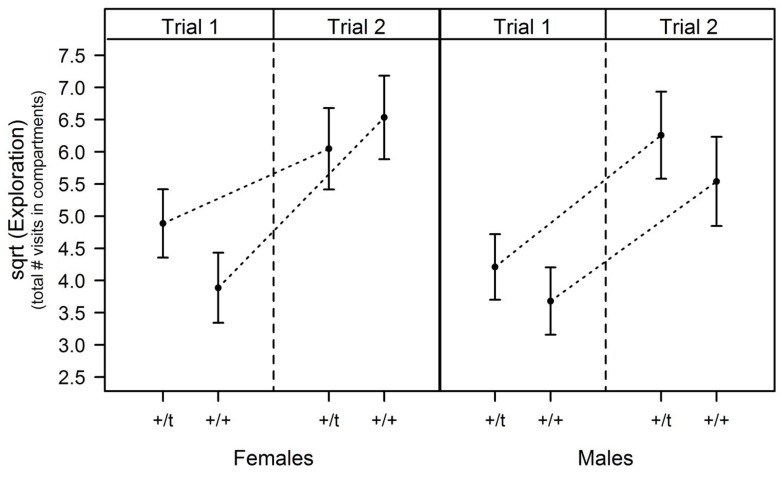
Effect of genetic background and sex on the propensity to form an exploratory routine: increase in exploratory behaviour (total number of visits to all compartments of the exploration apparatus) between the two trials (mean ± standard errors predicted by mixed effect model).

Genetic background, sex or their interaction did not have any significant influence on the propensity to form a routine observed in the boldness test, as measured by the number of visits to the cage containing soiled cat bedding or the number of visits to the cage containing clean cat bedding (Table 2). The analysis of the propensity to form an exploratory routine as measured by the increase in the total number of visits in the exploration test did not show an overall influence of sex or genetic background but a significant effect of the interaction of genetic background with sex (Table 2). Heterozygous +/*t* females were less prone to form an exploratory routine than +/+ females as they had a lower increase in their number of visits whereas there was no significant difference between +/*t* and +/+ males (Figure 3). When analysing the decrease in the time needed to visit all the compartments between the two replicates, sex, genetic background or their interaction did not show any significant effect on the formation of an exploratory routine (Table 2).

### Correlations between Personality Traits

**Figure 4:**
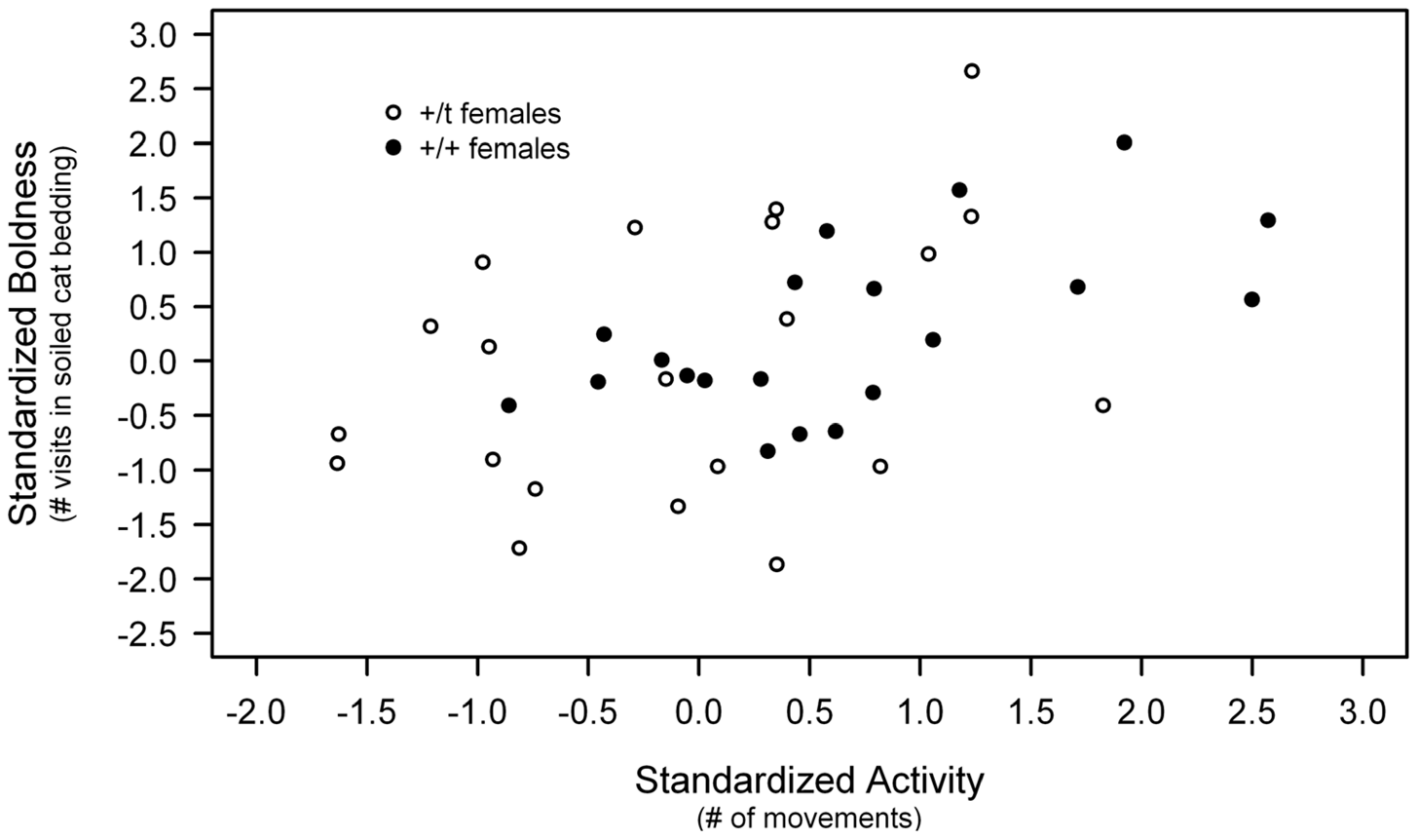
Activity - Boldness syndrome in females, according to genetic background.

The positive relationship between boldness and activity allowed us to define a behavioural syndrome in females but not in males (Table 3). More precisely, this relationship was significant in +/+ females whereas +/*t* females only showed a non-significant tendency to express it (Figure 4; Table 3).

**Table 3:** Correlations between personality traits showing individual consistency.

				Males						Females					
Behaviour pairs		All individuals		All genotypes		+/+		+/*t*		All genotypes		+/+		+/*t*	
		*Pearson r*	*p*	*Pearson r*	*p*	*Pearson r*	*p*	*Pearson r*	*p*	*Pearson r*	*p*	*Pearson r*	*p*	*Pearson r*	*p*
**Boldness**×**Activity**	# visits cat bedding×# movements	**0.31**	**0.005**	0.06	0.716	0.26	0.259	−0.23	0.312	**0.47**	**0.002**	**0.62**	**0.004**	0.37	0.101
**Boldness**×**Exploration**	# visits cat bedding×# visits	0.19	0.080	0.30	0.059	0.45	0.047	0.11	0.643	0.07	0.649	0.16	0.512	0.03	0.882
**Activity**×**Exploration**	# movements×# visits	0.16	0.147	0.14	0.383	0.35	0.131	0.05	0.831	0.14	0.396	0.26	0.274	0.07	0.763

Correlations remaining significant after Benjamini & Hochberg correction procedure are in bold.

### Food Consumption

**Figure 5:**
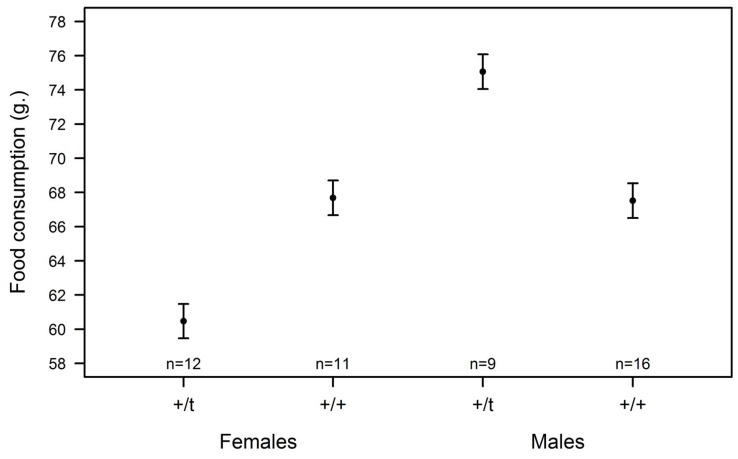
Influence of genetic background and sex on food consumption controlled for body weight (mean ± standard errors predicted by linear regression model).

Even though genetic background (*F*_1,46_ = 0.12, *p* = 0.73), sex (*F*_1,45_ = 3.05, *p* = 0.09), and body weight (*F*_1,44_ = 2.56, *p* = 0.12) did not show an overall influence on food consumption, the interaction between genetic background and sex had a marginally significant effect (*F*_1,43_ = 3.72, *p* = 0.06). Whereas +/+ males ate less than +/*t* males (+/+ males: 69.01±3.79 g., +/*t* males: 76.95±6.23 g.), the opposite was true in females, as +/*t* females ate less than +/+ females (+/*t* females: 61.02±2.59 g., +/+ females: 68.27±2.88 g.; Figure 5).

## Discussion

Our study demonstrated that laboratory reared female house mice of a genotype conferring a survival advantage under natural conditions expressed reactive-like behavioural traits favouring cautiousness and energy conservation. The longer living +/*t* females were less active, less prone to form an exploratory routine, and tended to ingest less food than the shorter living +/+ females.

Having a low activity level could have various positive effects on survival. First, decreasing activity can be beneficial for small rodents when facing predators relying on hearing or sight to detect prey [50]. Second, organismal maintenance requires partitioning of the available energy budget to different biological functions among which effector organs like skeletal muscles are responsible for much of the daily energy expenditure [51]. Within a given energy budget, an individual with a reduced activity can attribute a large part of its energy budget to other functions that could improve survival.

Our results on food consumption supported our energy-saving interpretation as +/*t* females showed a tendency to have a lower food intake than +/+ females. This could reflect a lower need for energy and/or a better capacity to save energy that could both favour survival when access to food is restricted or risky. Our results suggest that reactive individuals could decrease the frequency of their visits to feeding places compared to proactive individuals and may decrease the risk of being caught by predators when feeding. Moreover, research on rate of aging in rodents showed that mice fed with a 65% reduced diet improve their maximum life span by 51% compared to mice fed *ad libitum* [52]. Caloric restriction extends life span through mechanisms such as reduced oxidative damage [53]. This could also apply to +/*t* females and hence would partly explain their survival advantage over +/+ females that have a higher food consumption.

Moreover, +/*t* females were less prone to form an exploratory routine. Although reactive and proactive individuals have similar learning abilities, at least in birds, reactive individuals form routines slower than proactive individuals [9], [25]. This particularity, seen as a higher attentiveness to the environment, confers an advantage to reactive individuals as they can better adjust to sudden environmental changes than proactive individuals [7], [54], [55].

Conversely to other personality studies, we did not observe behavioural syndromes between most of the personality traits we assessed [5], [6]. We found a syndrome defined by a positive correlation between activity and boldness, such that the less active females were also the more cautious. However, this relationship was significant in +/+ females whereas +/*t* females only showed a tendency. Some studies have shown that behavioural syndromes are not ubiquitous, even within the same species. In three-spined sticklebacks (*Gasterosteus aculeatus*) the presence of behavioural syndromes depends on whether population characteristics favour suites of correlated behaviours [32], [56], [57]. The absence of behavioural syndromes in male house mice could thus be due to sex-specific behavioural optima.

The differences observed in the activity test are consistent with expected differences in energy demands due to milk production. Costs of lactation are very high in small rodents and increase with litter size [58]. Litter size is influenced by the *t* haplotype. On average +/*t* females have smaller litters than +/+ females, as whenever +/*t* females mate with +/*t* males their litter sizes are nearly halved due to the lethal homozygous effect of the *t* haplotype [59]. Thus a female’s expected average litter size should correlate with activity levels. Higher activity levels help to gather information about food to cover energetic needs during lactation. Consistent with this, we showed for non-breeding mice that +/*t* females had lower activity levels than did +/+ females. Fitness of +/*t* and +/+ females will on average be equal if +/*t* females compensate for smaller litters by producing more litters, which greater longevity would permit. This would contribute to maintaining the polymorphism in the population. Perrigo [60] showed that lactation strongly influences activity patterns of females, and that males were less active than females. We also found that males were less active than females.

The lack of difference in exploration and boldness between mice of different sexes and genotype suggests that balancing selection has resulted in a single optimal behavioural level for each, with no correlation between individual values for each traits. House mice in western Europe live commensally with humans and nearly always are found close to easily accessible food resources, and often in dense population [26], [38], suggesting that exploration to find new food patches may often be secondary to exploration to monitor social situations. Both males and females monitor the presence of conspecifics and defend their territories against intruders [61]. Similarly, boldness behaviour might be under strong balancing selection pressure reducing inter-individual variability, the raw material needed to evolve personalities.

Although our study provides interesting insights into personality traits associated with +/*t* females and survival differences, the causal relationship is unclear. The *t* haplotype, consisting of a third of chromosome 17, has had an independent evolutionary history from its wildtype counterpart for more than two million years [62]. Major Histocompatibility Complex genes are located within the four inversions comprising the *t* haplotype [63] and there is evidence that a gene influencing both male and female mate choice is also located within the *t* haplotype [20]. Genes influencing other traits, such as personality and/or survival, either additively or epistatically or through dominance, could be located within this region.

Behavioural studies like ours do not only help in understanding the *t* haplotype but also underline new questions related to life-history trade-offs and the evolution of animal personalities [13], [14], [64]. The rate-of-living theory postulates a negative association between life span and the rate of energy expenditure [65]. Thus two opposite strategies “live fast and die young” or “live slowly and die old”, define a fast-slow life-history continuum along which individuals can be ranked [66], [67], [68]. Our results give evidence that these two life-history strategies apply to the *t* complex, with +/+ females living extravagantly and +/*t* females living frugally. However, Wolf et al. [14] predicted an association between residual reproductive value and risk-related behaviours like exploration or boldness so that we could expect +/*t* females to be shyer and less explorative than +/+ females. However, our results fail to provide full empirical support to theory as only activity showed a clear association with the *t* haplotype and we did not find a strong relationship between activity and boldness.

Literature provides few examples reporting the influence of personality traits like activity, aggressiveness, and sociality on reproductive success or longevity [69], [70], [71] (see [72] for a review). Our study indicates that longer living house mice express reactive personality traits, demonstrating that longevity correlates with personality. However, as studies focusing on life-history productivity and personality are still missing in this species, we do not know if the expression of specific personality traits could also influence their reproductive success and/or tactics [13], [64].
