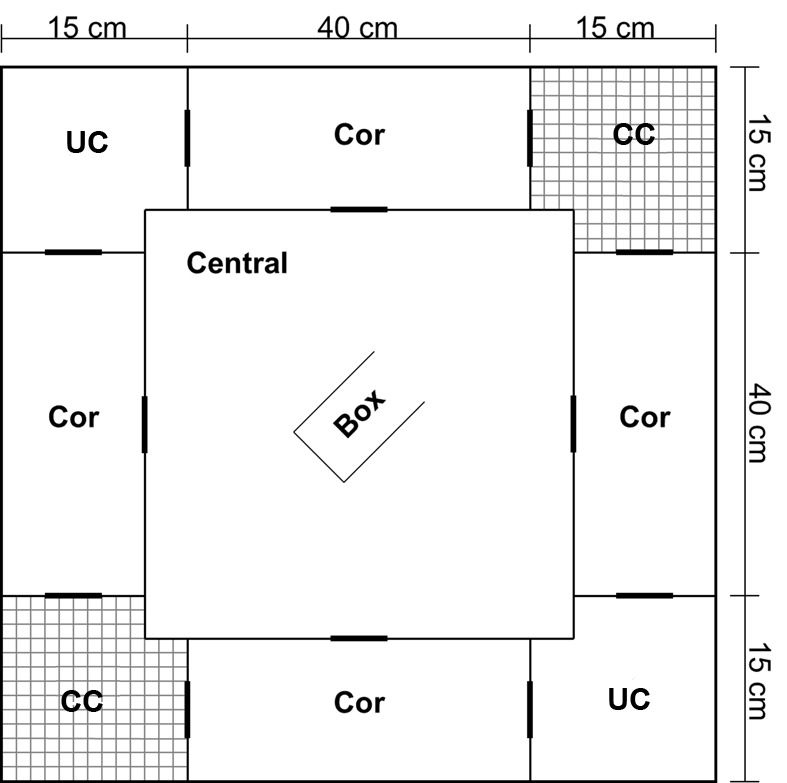# Correction: A Selfish Genetic Element Influencing Longevity Correlates with Reactive Behavioural Traits in Female House Mice (*Mus domesticus*)

**DOI:** 10.1371/annotation/475466bd-f4ff-433e-b2e0-67d071f0019a

**Published:** 2014-01-03

**Authors:** Yannick Auclair, Barbara König, Anna K. Lindholm

In Figure 1, the labels on the corners are incorrect. LC should be UC and DC should be CC. Please see the corrected Figure 1 here: 

**Figure:**